# Smart snacks in universities: possibilities for university vending

**DOI:** 10.34172/hpp.2020.58

**Published:** 2020-11-07

**Authors:** Georgianna Mann, Laurel Greenway Lambert, Kritika Gupta, Megan Partacz

**Affiliations:** ^1^Department Nutrition and Hospitality Management, 220 Lenoir Hall, P.O. Box 1848, University of Mississippi, University, MS 38677, United States; ^2^University of Memphis Dining, University of Memphis, TN, 38152, United States

**Keywords:** United States, Snacks, Universitie, Healthy, Sodium, Iron

## Abstract

**Background:** The study goal was to evaluate the nutritional impact of a healthy snack intervention on a southern university campus.

**Methods:** This quasi-experimental study was conducted during the fall 2017 semester weekly for 14 weeks in a large southern U.S. university. For the intervention, half of vending snacks in four campus residential halls (housing from 216 to 361 students) were substituted with snacks complying with federal Smart Snacks in School nutrition standards for K-12 schools. For analysis, data from the Nutrition Facts labels of 14 vending machines or from manufacturer’s websites was collected by trained graduate and undergraduate researchers.

**Results:** On average, for each Smart Snack sold, there was a statistically significant reduction of 99.38 calories (CI=42.32, 156.43), 4 g saturated fat (CI = 2.23, 5.75), and 10.06 g of sugar(CI=2.92, 17.20). An average reduction of 41.88 mg in sodium and an increase of 0.81g in fiber was also found, but was not statistically significant. There was a significant difference (t(16)=3.02, P < 0.025, 95% CI = 10.77, 55.79) between the Quality Score of Smart Snacks (M=59.13,SD= ± 36.50) and that of non-compliant snacks (M=25.85, SD= ± 24.72).

**Conclusion:** The nutritional impact with even a 50% Smart Snack replacement is promising. Many available comparable snacks mimic the mouthfeel, taste, and appearance of their original full-fat, full-sodium, and full-sugar counterparts. Including healthier snack choices in vending machines may be a viable option for universities to transform the campus eating environment.

## Introduction

In the United States, the South continues to lead the nation with some of the highest obesity rates of more than 30% in adults.^1^ These obesity rates are alarming as obesity is a risk factor for chronic disease, type II diabetes and cancer,^2,3^ but also contributes to increased health care costs.^4^

Individual lifestyle habits, including diet and physical activity, are major determinants of weight status.^5^ These habits are often formed in childhood and continue into adulthood. Young adulthood, when many attend college, is a critical life stage for developing food behaviors that could direct many diet and physical activity behaviors held throughout their adult lives.^6^ As students, young adults start establishing independence, including food-related choices. While current American dietary trends do not comply with U.S. Department of Agriculture (USDA) dietary guidelines,^7^ on average university student diets poorer than that of the general adult population.^8^ It is also during this time when many students may encounter the well-known ‘freshman 15’: an unintentional weight gain during their first year in college.^9^

The food environment, in particular, plays a large role in food-related behaviors and often drives purchase decisions particularly in school settings.^10^ The food environment of many universities is typically found to host casual eateries, fast food restaurants, and snack foods from vending machines.^11^ Snacking behavior, where snacks are defined as food items consumed outside of culturally defined meals, has more recently become the focus of research in eating behaviors.^12^ Snacking behavior has increased, and most adults consume snacks daily.^13^ Snacking has been shown to contribute approximately one-quarter of daily caloric intake with 80% of college students consuming snacks daily, contributing to increased consumption of nutrient-devoid and energy-dense foods.^14^

While snacks are an opportunity to increase nutrient intake, most college students select snacks based on price, convenience, taste, accessibility, peer influence and occupied schedules.^14-17^ As reported, the most notable contributor to snacks low in nutritional value and high in solid fats and added sugars are the readily available vending machines on college campuses.^18^ Not only are these snacks low in nutrient density, but many packages contain more than one serving. With a large percentage of students residing on university campuses, these snacks are easily accessible through vending machines in residence halls and academic buildings. This has resulted in snacking behavior, particularly when snacks are of poor nutritional quality, that can negatively affect the overall dietary patterns.^19,20^

Most of the food and nutrition policies have concentrated their effort to improve the nutritional outcome of school students. However only a few studies have raised the importance of having a fixed nutrition policy for university food environment. Lambert and Joung^16^ assessed the snacks and beverages sold in a southern U.S. university vending machines and reported that only 2% of the snacks could be categorized as Smart Snacks which included baked potato chips, granola bars, and nuts. Shi et al^21^ conducted a cross-sectional study in an Australian university and reported a poor compliance of food outlets and vending machines with more than half of the available packaged choices being sugary drinks. The same study raised concern over not having a policy in place for healthier university food environments. Another United Arab Emirates based qualitative study acknowledge the poor nutritional quality of food products in university vending machines and advocated for improvement of nutritional quality of vending machine food products.^17^ To positively influence the food choices of vending machine consumers and to modify the food environment in the university, snack-based interventions have been suggested.^22^

Previous studies with snack-based interventions have shown positive outcomes when interventions include nutrition education, signage, and reduced prices for healthier items.^23^ However, the impact of increasing the availability of healthy products in vending machines on snack selection, along with a multi-level intervention, has not widely been addressed despite the potential for creating positive dietary change. The present study investigated the nutrient differences resulting from providing healthier snacks in vending machines located in residential halls and the potential for improved nutrient intake for students living in campus residential halls. This study was part of a larger quasi-experimental study that examined the impact of a multi-level nutrition intervention on increasing the selection of healthier snack items. Outcomes data of snack selection provided from the larger study will be used for the analysis.

## Materials and Methods

### Study sample

This quasi-experimental study was conducted in a large southern United States university during the fall 2017 semester weekly for 14 weeks. This study examined the nutrient content of selected snacks in 14 vending machines in four residential halls (housing from 216 to 361 students) on campus. This research is part of a larger study in which 4 nutrition interventions were applied. Halls were selected based on majority freshmen occupancy, since this group had recently graduated from high school and had the greatest chance of being familiar with *Smart Snacks.* Additionally, halls had comparable male-female ratios, comparable numbers of vending machines, and were located on the main campus. Access to halls and living quarters required a student ID, therefore limiting who had access to the vending machines. At the time of the study, 55% of residents were female and majority freshmen (89%).

### Intervention

Consistent in four selected residential halls was an intervention where 50% of all snack foods met *Smart Snacks in School* standards. These standards were mandated with the Healthy, Hunger Free Kids Act of 2010 and apply to all K-12 schools participating in the National School Lunch Program.^24^ When implemented in 2014, *Smart Snack* standards included restrictions on calories (≤ 200), fat (≤ 35% calories), saturated fat (< 10% calories), sodium (≤ 230 mg), and sugar (≤ 35% by weight). The other 50% of the snacks placed in the vending machines were determined by the vendor and were previously available, popular, and familiar to students. These snacks will hereafter be referred to as “non-compliant” (NC) snacks.

### Procedure

Trained graduate student researchers collected data from 14 vending machines in four residential halls during the fall 2017 semester weekly for 14 weeks. The research team created an observational spreadsheet based on the vendor’s visual representation of a vending machine’s offerings called a Plan-O-Gram as shown in similar vending audit studies.^25^ Vendor employees stocked the same items and followed the same item placement in vending machines for consistency. Trained graduate student researchers accompanied vendors during weekly restocking to record the number of products on hand, the number of products expired and therefore discarded, and the number of products restocked. Prior to discarding any snacks, researchers used number of items on hand minus number of items restocked to obtain sales data. Lead researchers reviewed and compared completed audit tools to ensure validity.

Trained graduate and undergraduate researchers entered data collected from the Nutrition Facts labels, either from the snacks directly or from manufacturer’s websites, for analysis.

### Research design

**Figure 1:**
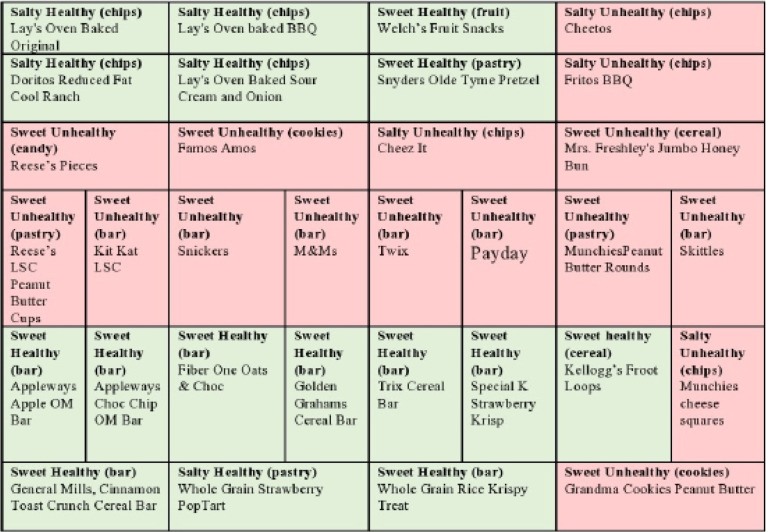
Plan-O-Gram for 32-item snack machine.

The research team, university contractual services manager, and vending company worked together to implement a 50% *Smart Snacks* intervention in the targeted vending machines. The Plan-O-Gram for the 32-item snack machine is shown in Figure 1.

### Statistical analysis

Means were computed for the nutrient content of 16 *Smart Snacks* used in this study and for 16 vendor selected NC snacks (Figure 1). The research team computed additional information including change in nutrient adequacy ratio (NAR), nutrients to maximize score (N_max_), Nutrients to minimize score (N_min_) and quality scores (QS) as described by Byrd-Bredbenner et al^18^ and as explained in Table 1. N_max_ is the average of recommended nutrients, i.e. dietary fiber, protein, Vitamin D, calcium, iron, and potassium, whereas N_min_ is the average of restricted nutrients, i.e. saturated fat, cholesterol, sodium, and total sugars. A QS of 100 indicates that N_max_ and N_min_ are equal. When N_max_ > N_min_, QS will be over 100 and when N_max <_ N_min_, QS will be under 100. A food product with a higher QS indicates that the product is high in desirable nutrients. Independent samples t-test was used to compare the mean differences of nutrients between NC and *Smart Snacks*. Independent samples *t* test was also used to compare the mean differences of NAR between NC and *Smart Snacks* . Significance is reported as *P*  < 0.05, *P* < 0.025, and *P* <0.001. Equal variances were assumed for conducting independent samples *t* test. The results for average nutrients and NAR are reported as mean ± SD. The IBM SPSS Statistics, version 26 (Chicago, IL, USA) was used to analyze the data for the study.

## Results

### Selection of Smart Snacks 

For one academic semester, students purchased a total of 16,822 snacks from the 14 modified vending machines. Of these, 10,849 were NC and 5,973 were *Smart Snacks*. Vending employees discarded 300 NC snacks and 514 *Smart Snacks* as they were past printed “sell by” or “best by” dates. Average sales per week were approximately 775 NC snacks and 427 *Smart Snacks* .

### Average nutrient content

A statistically significant reduction in energy content between NC snacks (M=236.25, SD=109.42) and *Smart Snacks* (M=136.88, SD=22.72) was found (*t* (16) = -3.56, *P* < 0.001, 95% CI = 42.32, 156.43). Among restricted nutrients, a significant reduction (*t* (16) = -4.68, *P* <0.001, 95% CI = 2.23, 5.75) in saturated fat content was reported between NC (M=4.53, SD = 3.39) and *Smart Snacks* (M=0.53, SD=0.46). Total sugar content was also significantly less (*t* (16)=-2.88, *P* < 0.001, 95% CI=2.92, 17.20) in *Smart Snacks* (M=7.75, SD=4.34) as compared to NC snacks (*M* =17.75, *SD* =13.26). The average dietary fiber content in *Smart Snacks* (M=1.94, SD=2.21) was higher than that found in NC snacks (M=1.25, SD=1.13) but was statistically not significant (*t* (16)=1.34, *P* =0.19, 95% CI = -2.05, 0.42) (Table 2).

### Nutrient adequacy ratio

There was a significant difference (*t* (16)= -2.88, *P*  < 0.025, 95% CI = -0.34, -0.06) in NAR of total sugar between NC snacks (M=-0.36, SD= ± 0.27) and *Smart snacks* (M=0.15, SD*=* ± 0.09) and that of saturated fat (*t* (16)= -4.68, *P* <0.001, 95% CI = -0.34, -0.06) between NC snacks (M=-0.23, SD= ± 0.170) and *Smart Snacks* (M=0.03, SD= ± 0.02) (Table 3). Though statistically non-significant, there was a reduction in mean NARs of cholesterol (*t* (16)= -0.37, *P* =0.712, 95% CI = -0.01, 0.004), sodium (*t* (16)= -0.08, *P* =0.146, 95% CI = -0.08, 0.01), and total carbohydrates (*t* (16)= -0.67, *P* =0.511, 95% CI = -0.09, 0.04), as well. The N_min_ of NC snacks (M=0.17, SD= ± 0.10) was significantly higher (*t* (16) = -4.39, *P* <0.001, 95% CI = -0.16, -0.06) as compared to *Smart Snacks* (M=0.07, SD= ± 0.03), and a lower (M=0.03, SD*=* ± 0.02) but non-significant N_max_ (*P* =0.501) as compared to that of *Smart Snacks* (MD=0.04, SD*=* ± 0.03).

### Quality score

**Figure 2:**
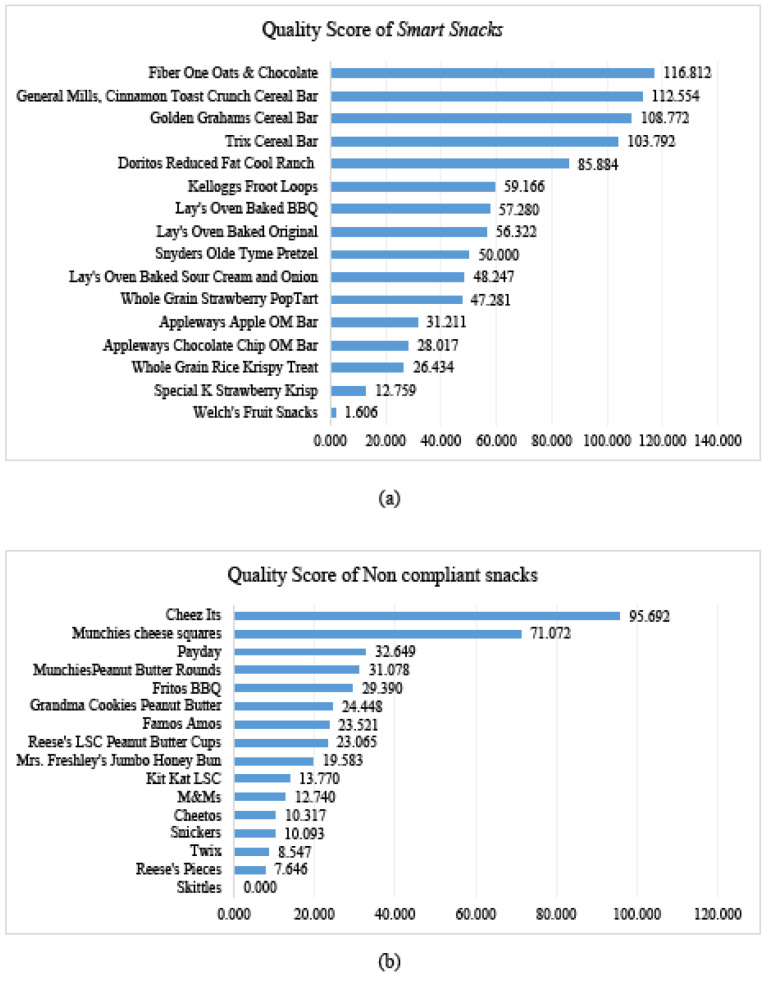
(a) Quality score of Smart snacks (b) Quality score of Non-compliant snacks

There was a significant difference (*t* (16)= 3.02, *P* <0.025, 95% CI = 10.77, 55.79) between the QS of *Smart Snacks* (M=59.13 ± SD = 36.50) and that of NC snacks (M=25.85, SD*=* ± 24.72). As mentioned earlier, a QS over 100 indicates that N_max_ is higher than N_min_. Four *Smart Snacks* and none of the NC snacks had a QS over 100, indicating poor nutritional content of NC snacks, and justifying the nutrient appropriateness of *Smart Snacks* . A total of nine *Smart Snacks* and two NC snacks had a QS of 50 or higher (Figure 2).

## Discussion

As snacking increases and is becoming a common food consumption pattern, attention towards the nutrients found in snacks should increase as evident by this research. This is especially important considering young adulthood can drive future dietary behaviors. Taste is often the prime factor when determining snack choices.^26^ Therefore, importance must be placed on offering students an increased number of high quality score snacks which students find acceptable and would purchase.

### Selection of Smart Snacks

The nutrient content of snacks purchased from 14 snack vending machines were analyzed and nutritional comparisons were made between the *Smart Snacks* and NC snacks commonly found in the vending machines. Overall, and not surprisingly, student selection of NC snacks was higher than that of *Smart Snack* items, as also shown by other researchers.^27^ Many *Smart Snacks* were newly introduced to machines, while NC snacks were selected by the vendor based on popularity as reflected in sales data. Research on *Smart Snacks* implementation in K-12 schools has shown a similar trend, where snack sales declined upon implementation, yet sales increased after the initial year of standard compliance.^28^ This fear of revenue loss is a primary barrier to implementing healthier nutrient standards both at a vendor and institutional level.^29^ Research has shown that this profit reduction can be mitigated in the following years and when competition from outside venues are minimized.^30^ In the context of this study, the profits lost by the price reduction strategy employed here were compensated by the University’s wellness program to encourage vendor participation.

Additionally, more waste was generated by *Smart Snacks* relative to NC options. As an increasing number of food companies are modifying their products in response to consumer trends and demands, use of natural preservatives or reliance on packaging alone for preservation is observed. This change may appeal to consumer interests but can decrease shelf life and generate more product waste. Sodium in particular plays a key role in food preservation, and it is possible that the lower sodium in *Smart Snacks* options shortens shelf life.^31^ Another factor to be considered is that often these modified versions of common snacks are lower in fat. As a preservative, fat can help to prevent staling of snack foods, especially chips.

The high number of wastes among *Smart Snacks* could also have been mitigated by the vendor’s practice of setting par levels for stocking vending machines. Par levels that may have been appropriate for the NC snacks, which sell at a quicker pace, may have been set too high for *Smart Snacks* resulting in many going out of date. Vendors incorporating snacks with similar nutrient guidelines as *Smart snacks* will need to adjust or allow more flexibility in their vending machine stocking policies.

### Nutrient composition

While *Smart Snacks* equaled to about 1/3 of overall snack selection by students, the potential nutritional benefit with even a 50% *Smart Snack* replacement is promising. Beyond the caloric reduction, a *Smart Snack* vending intervention could provide a notable reduction in sodium. On average, by using the snacks shown in this study, (substituting *Smart Snacks* for NC snacks), a considerable reduction in sugar intake could occur, which is undesirably high in current diets of Americans.^7^ Choosing *Smart Snacks* over NC snacks also leads to a significant reduction of sugar and saturated fat, and a lowering of cholesterol and sodium in selected snacks. These nutrients are currently present in high amounts in American diets and are among the major contributors of cardiovascular disease.^32^ None of the NC snacks had a QS of 100 or greater, indicating that the campus wide vending machines are primarily stocked with nutritionally poor snacks.

Some of the nutritional differences between *Smart Snacks* and other snack choices could be due in part to the use of appropriate portion sizes for *Smart Snacks*. Over the past five decades, portion sizes of snacks, restaurant meals, and beverages have increased to often far exceed federal standards.^33^ Research has demonstrated that even independent of package size, college students are not proficient at detecting appropriate portion sizes.^34^ Compounding this issue, container sizes often drive consumption of products, including snacks shown here reaching upwards of 590 calories for a “snack” offered in a vending machine.^35^ In light of this, the Food and Drug Administration has changed the Nutrition Facts label to account for total calories in a package as well as altering the serving sizes of commonly overeaten foods such as ice cream.^36^ It should be pointed out that the QS is just one measure used to gauge the nutritional adequacy or density of a food. For example, while *Welch’s fruit snacks* met the nutritional requirements for inclusion as a *Smart Snack* the lack of sufficient calcium, Vitamin D, or protein resulted in a low QS. On the other hand, in the NC snack *Cheez Its* , with the presence of calcium, iron and potassium increased the QS to a relatively high score The QS of a snack is more of a numerical approach towards analyzing the healthfulness of a snack and, though important, it should not be considered as the sole factor in determining the nutritional value of a snack. While making a judgment on purchasing snacks, a comprehensive picture of individual nutrients, NAR and QS should be considered.

*Smart Snacks* offer an improvement in nutritional quality but are not, alone the key to a healthy diet. Research has established that by increased access to healthy foods and decreasing the availability of less-nutritious food, can prove to be effective in improving eating behavior and hence the health outcomes of individuals.^37,38^ Only 50% intervention was used in the current study, and it produced significant changes; higher intervention can be used to strengthen the effect.

This study, while telling, has limitations. This study was completed in one university in the southern region of the United States and, as such, results cannot be generalized. Data were collected only in residential halls and not campus wide. This study was completed in partnership with the campus wellness initiative and provided reimbursements to the vendor to encourage participation. In fall of 2017, waste accounted for $565.50 over the entire semester. The vendor was paid $1,530 throughout the semester to account for differences in typical sales. Another limitation is that certain variables like demographics of the students in the halls or the number of students in each hall or the events like football matches were not controlled while conducting statistical analyses. A survey of students with their input as to what *Smart Snacks* should be in vending could have been conducted prior to beginning the study to strengthen the potential of snack sales. The study was only completed during one semester, which could limit the potential results seen in a longer study.

### Policy questions

This study lends some policy insight. It is not uncommon for vendors to have “pouring rights” contracts on K-12 and higher education campuses which prohibits none but specific brand items to be sold on campus.^39^ These can create a marketing pipeline, where students are consistently exposed to persistent and persuasive industry advertisements enticing students to purchase various snacks and beverages. Implementation of healthier options in vending machines has been shown to be financially feasible, where healthier snacks can be offered without a substantial decline in sales.^40^ Providing smarter snack choices may come at a perceived cost for contractual services, but the benefits for students could be far more valuable.^30^ The overarching question is; are universities willing to set policies directing the offering of healthier choices on campus, which could impact finances, in order to provide a healthier food environment for students and the university community on whole.

Universities have been proactive at creating smoke-free campus environments with positive reception.^41^ It is now time for universities to address the campus food environment and starting with vending machines could be impactful. Previous work has shown the recommended steps needed to begin a healthy vending snack change.^42^ Critical pieces of the process include identification of nutrition standards for adherence, seeking involvement from contractual services and other university stakeholders, and creating and maintaining a formative evaluation strategy.^40,42^

This study sheds light on the potential small snack interventions can have for student health. Students are primarily driven by taste and conveniences when selecting a snack.^43-45^ By making the smart snack the easy, convenient, and preferable, it may assist students in reducing sodium, fat, and added sugars.

## Acknowledgements

We want to thank Kathy Tidwell, Manager of Contractual Services, and Mariana Jurss registered dietitian with university wellness for their assistance during this study. We also want to thank the NHM graduate students who collected data.

## Funding

This study was funded by the university’s wellness program.

## Competing interests

None to declare.

## Ethical approval

This study was deemed exempt by the University of Mississippi Institutional Review Board as this component of the study did not involve any interaction with human subjects.

## Authors’ contributions

GM and LGL formulated the project, and was involved in data collection, analysis, and manuscript development. KG did data analysis and helped in manuscript development. MP was involved in data collected and helped in data analysis and manuscript development.

**Table 1:** Steps to calculate Nutrient Adequacy Ratios and Quality Scores

**Step 1 Calculate NAR** Nutrient Adequacy Ratio (NAR) of nutrient X=Amount of nutrient X in food productRecommended daily value of the nutrient X	
Example Fiber One Oats & Chocolate (40g)
Saturated Fat NAR	1.5 g saturated fat / 20 g Daily Value for saturated fat = 0.075
Cholesterol NAR	0 mg cholesterol / 300 mg Daily Value for cholesterol = 0
Sodium NAR	95 mg sodium / 1500 mg Daily Value for sodium = 0.06
Carbohydrate NAR	29 g carbohydrate / 130 g Daily Value for carbohydrate = 0.22
Dietary Fiber NAR	9 g dietary fiber / 25 g Daily Value for dietary fiber = 0.36
Total sugar NAR	9 g total sugar / 50 g Daily Value for total sugar = 0.18
Protein NAR	2 g protein / 150 g Daily Value for protein 0.01
Vitamin D NAR	0 IU Vitamin D / 600 IU Daily Value for vitamin D = 0
Calcium NAR	140 mg calcium / 1000 mg Daily Value for Calcium = 0.14
Iron NAR	0.80 mg iron / 18 mg Daily Value for iron = 0.04
Potassium NAR	0 mg potassium / 4700 mg Daily Value for potassium = 0
**Step 2 Calculate Nutrients to maximize score N**_max_ Example Fiber One Oats & Choc (40g) $N\;\max=\frac{Diertary\;fiber\;NAR+\mathrm{Pr}otein\;NAR+Vita\min\;D\;NAR+Calcium\;NAR+Iron\;NAR+Potassium}{6}=0.09$	
**Step 3 Calculate Nutrients to minimize score N**_min_ Example Fiber One Oats & Choc (40g) $N\;\min=\frac{Saturated\;NAR+Cholesterol\;NAR+Sodium\text{​}\;NAR+Total\;sugar\;NAR}{4}=0.08$	
**Step 4 Calculate Quality Score** Example Fiber One Oats & Chocolate (40g) $Quality\;Score=\frac{N\max}{N\min}\;X100=\frac{0.09}{0.08}=116.81$	

**Table 2:** Average Nutrient Content of NC snacks and *Smart Snacks*

	**NC snacks**		***Smart Snacks***		**t-test for equality of means**		**95% CI for mean differences**
	**Mean ± SD**	**Minimum, Maximum**	**Mean ± SD**	**Minimum, Maximum**	**t**	**Mean difference**	
Energy (kcal)	236.25 ± 109.42	130, 590	136.88 ± 22.72	90, 200	-3.56***	-99.38	42.32, 156.43
Total fat (g)	11.09 ± 5.66	2, 28	3.06 ± 1.38	0, 10	-5.52***	-8.03	5.06, 11.00
Saturated fat (g)	4.53 ± 3.39	0, 14	0.53 ± 0.46	0, 1.5	-4.68***	-4.00	2.23, 5.75
Cholesterol (mg)	1.54 ± 3.15	0, 40	0.63 ± 1.71	0, 5	-1.00	-0.91	-0.97, 2.80
Sodium (mg)	171.88 ± 121.86	0, 800	123.13 ± 46.58	25, 210	-1.50	-48.75	-17.86, 115.36
>Total Carbohydrates (g)	29.00 ± 15.48	0, 76	26.25 ± 5.46	19, 38	-0.67	-2.75	-5.63, 11.13
Dietary fiber (g)	1.25 ± 1.13	0, 4	1.94 ± 2.21	0, 9	1.34	0.81	-2.05, 0.42
Total sugar (g)	17.75 ± 13.26	0, 52	7.75 ± 4.34	0, 15	-2.88**	-10.06	2.92, 17.20
Protein (g)	2.81 ±1.83	0, 16	1.81 ± 0.66	0, 3	- 2.05	-1.00	0.01, 1.99
Vitamin D (IU)	0.68 ± 1.84	0, 6.5	± 0.50	0, 2	- 1.17	-0.56	-0.42, 1.53
Calcium (mg)	35.68 ± 36.50	0, 340	59.44 ± 79.83	0, 200	1.08	23.76	-68.58, 21.05
Iron (mg)	1.37 ± 1.28	0, 4	1.13 ± 1.14	0, 4.5	- 0.54	-0.23	-0.65, 1.11
Potassium (mg)	63.75 ± 58.64	0, 530	58.94 ± 85.47	0, 250	-0.53	-4.81	-48.11, 57.73

*Indicates significant differences noted by an independent-samples t-test (P < 0.05), ** P < 0.025, *** P < 0.001.

**Table 3:** Nutrient adequacy ratio of different nutrients for non-compliant snacks and *Smart Snacks*

	**NC snacks**		***Smart snacks***		**t-test for equality of means**		**95% CI for mean differences**
	**Mean ± SD**	**Minimum, Maximum**	**Mean ± SD**	**Minimum, Maximum**	**t**	**Mean difference**	
**N**_max_	0.03 ± 0.02	0.00, 0.08	0.04 ± 0.03	0.00, 0.09	0.68	0.01	-0.01, 0.02
NAR Total carbohydrates	0.22 ± 0.12	0.12, 0.54	0.23 ± 0.09	0.15, 0.29	-0.67	-0.02	-0.09, 0.04
NAR Dietary fiber	0.05 ± 0.05	0.00, 0.16	0.08 ± 0.09	0.00, 0.36	1.34	0.03	-0.02, 0.08
NAR Protein	0.02 ± 0.01	0.00, 7.00	0.01 ± 0.00	0.00, 0.02	-2.11*	-0.01	-0.01, 0.00
NAR Vitamin D	0.00 ± 0.003	0.00, 0.01	0.00 ± 0.00	0.00, 0.00	-1.24	-0.00	-0.003, 0.00
NAR Calcium	0.04 ± 0.04	0.00, 0.15	0.06 ± 0.08	0.00, 0.20	1.08	-0.29	-0.02, 0.07
NAR Iron	0.08 ± 0.07	0.00, 0.22	0.06 ± 0.06	0.00, 0.25	-0.54	-0.01	-0.06, 0.04
NAR Potassium	0.01 ± 0.01	0.00, 0.03	0.01 ± 0.02	0.00, 0.05	-0.19	-0.00	-.001, 0.01
**N**_min_	0.17 ± 0.10	0.05, 0.40	0.07 ± 0.03	0.02, 0.12	-4.39***	-0.11	-0.16, -0.06
NAR Saturated fat	0.23 ± 0.17	0.05, 0.70	0.03 ± 0.02	0.00, 0.08	-4.68***	-0.20	-0.29, -0.11
NAR Cholesterol	0.00 ± 0.01	0.00, 0.03	0.00 ± 0.01	0.00, 0.02	-0.37	-0.00	-0.01, 0.004
NAR Sodium	0.11 ± 0.08	0.01, 0.30	0.08 ± 0.03	0.02, 0.14	-1.49	-0.03	-0.08, 0.01
NAR Total sugar	0.31 ± 0.30	0.00, 0.90	0.17 ± 0.13	0.00, 0.30	-2.88**	-0.20	-0.34, -0.06
**Quality Score**	25.85 ± 24.72	0.00, 95.69	59.13 ± 36.50	1.61, 116.81	3.02**	33.28	10.77, 55.79

NAR = Nutrient Adequacy Ratio; N_max =_ Nutrients to maximize score; N_min =_ Nutrients to minimize score. *Indicates significant differences noted by an independent-samples t-test (P < 0.05), ** P < 0.025, *** P < 0.001.
